# The coronavirus disease pandemic among adult congenital heart disease patients and the lessons learnt – results of a prospective multicenter european registry^☆^

**DOI:** 10.1016/j.ijcchd.2022.100428

**Published:** 2022-11-21

**Authors:** Francisco Javier Ruperti-Repilado, Helmut Baumgartner, Berto Bouma, Judith Bouchardy, Werner Budts, Laurence Campens, Massimo Chessa, Maria Jesús del Cerro Marin, Harald Gabriel, Pastora Gallego, Elvira Ana González, Annette Schophuus Jensen, Magalie Ladouceur, Christopher Lockhart, Berta Miranda-Barrio, Marielle Morissens, Eduardo Moreno Escobar, Agnès Pasquet, Joaquin Rueda Soriano, Annemien Elise van den Bosch, Heleen Berdina van der Zwaan, Daniel Tobler, Matthias Greutmann, Markus Schwerzmann

**Affiliations:** a Centre for Congenital Heart Disease, Cardiology, University Hospital Inselspital, University of Bern, Switzerland; b Department of Cardiology III - Adult Congenital and Valvular Heart Disease, University Hospital Muenster, Germany; c Department of Cardiology, Academic Medical Centre, Amsterdam, Netherlands; d Department of Cardiology and Cardiac Surgery, University Hospital Lausanne, Lausanne, Switzerland; e Division of Cardiology, University Hospital Geneva, Geneva, Switzerland; f Congenital and Structural Cardiology, University Hospitals Leuven, Belgium; g Department of Cardiovascular Sciences, Catholic University Leuven, Belgium; h Cardiology Department, Ghent University Hospital, Ghent, Belgium; i ACHD-Unit. Pediatric and Adult Congenital Heart Centre, IRCCS-Policlinico San Donato, San Donato Milanese, Milan, Italy; j Vita Salute San Raffaele University, Milan, Italy; k Pediatric Cardiology & GUCH Centre, Ramon y Cajal University Hospital, Spain; l Medical University of Vienna, Department of Cardiology, Adult Congenital Heart Disease Program, Vienna, Austria; m Adult Congenital Heart Disease Unit, Department of Cardiology, Hospital Universitario, Virgen Del Rocio, Instituto de BioMedicina de Sevilla (IBIS) and CIBERCV, Sevilla, Spain; n La Paz University Hospital, Madrid, Spain; o Department of Cardiology, Rigshospitalet, Copenhagen University Hospital, Denmark; p Université de Paris, Hôpital Européen Georges Pompidou, AP-HP, Adult Congenital Heart Disease Unit, Centre de Référence des Malformations Cardiaques Congénitales Complexes, M3C. Inserm U970, Paris Centre de Recherche Cardiovasculaire, Paris, France; q Department of Cardiology, Belfast Health and Social Care Trust, BT126BA, United Kingdom; r Unitat Integrada de Cardiopaties Congènites de L'Adolescent I L'Adult Hospital Vall D'Hebron-Sant Pau. Servei de Cardiologia. Vall D’Hebron Hospital Universitari, Vall D’Hebron Barcelona Hospital Campus, Passeig Vall D'Hebron 119-129, 08035, Barcelona, Spain; s CHU Brugmann, Brussels, Belgium; t Inter-Center Unit for Congenital Heart Disease in Adults of Granada. Virgen de Las Nieves-Clínico San Cecilio University Hospitals. IBS, Granada, Spain; u Pôle de Recherche Cardiovasculaire, Institut de Recherche Expérimentale et Clinique, Université Catholique de Louvain and Divisions of Cardiology and Cardiothoracic Surgery, Cliniques Universitaires Saint-Luc, Brussels, Belgium; v Adult Congenital Heart Disease Unit, Department of Cardiology, Hospital Universitari I Politècnic La Fe and CIBERCV, València, Spain; w Erasmus Medical Centre, Rotterdam, Netherlands; x University Medical Centre Utrecht, Netherlands; y Division of Cardiology, University Hospital of Basel, University of Basel, Switzerland; z University Heart Centre, Department of Cardiology, University of Zurich, Switzerland

**Keywords:** Adult congenital heart disease, Coronavirus disease 2019, Risk stratification, Outcomes

## Abstract

**Background:**

At the beginning of the COVID-19 pandemic, professionals in charge of particularly vulnerable populations, such as adult congenital heart disease (ACHD) patients, were confronted with difficult decision-making. We aimed to assess changes in risk stratification and outcomes of ACHD patients suffering from COVID-19 between March 2020 and April 2021.

**Methods and results:**

Risk stratification among ACHD experts (before and after the first outcome data were available) was assessed by means of questionnaires. In addition, COVID-19 cases and the corresponding patient characteristics were recorded among participating centres. Predictors for the outcome of interest (complicated disease course) were assessed by means of multivariable logistic regression models calculated with cluster-robust standard errors. When assessing the importance of general and ACHD specific risk factors for a complicated disease course, their overall importance and the corresponding risk perception among ACHD experts decreased over time. Overall, 638 patients (n = 168 during the first wave and n = 470 during the subsequent waves) were included (median age 34 years, 52% women). Main independent predictors for a complicated disease course were male sex, increasing age, a BMI >25 kg/m2, having ≥2 comorbidities, suffering from a cyanotic heart disease or having suffered COVID-19 in the first wave vs. subsequent waves.

**Conclusions:**

Apart from cyanotic heart disease, general risk factors for poor outcome in case of COVID-19 reported in the general population are equally important among ACHD patients. Risk perception among ACHD experts decreased during the course of the pandemic.

## Highlights

• Overall risk perception among ACHD experts decreased over time.
• Apart from cyanotic heart diseases, general risk factors for poor outcome in case of COVID-19 in the general population are also important in ACHD.
• Our registry helped improving risk-stratification of ACHD patients during the pandemic.

## Introduction

In March 2020, the World Health Organization (WHO) declared the coronavirus disease 2019 (COVID-19) a pandemic. Back in those first weeks of the pandemic, the whole community of health care providers was confronted with a new and challenging situation. In the beginning, only very limited data on outcomes were available for the general population. While pre-existing cardiovascular disease was identified as an important predictor for a dismal disease course in case of infection with the novel severe acute respiratory syndrome coronavirus 2 (SARS-CoV-2), it remained unclear whether this association applied to the mostly young adults with congenital heart disease (ACHD) in a similar manner [[1], [2], [3], [4], [5], [6], [7]].

To facilitate risk stratification, we started a prospective multicenter European registry of COVID-19 cases among ACHD patients. Our aims were: 1.) to assess the perceived risk in case of COVID-19 in different types of ACHD patients among ACHD-experts and 2.) to collect real-time data on outcomes of affected ACHD patients among the participating centres.

Back in March and April 2021 we published the COVID-19 outcome in ACHD patients infected during first wave of the pandemic and the results of a questionnaire assessing risk stratification habits among ACHD experts, respectively [8,9]. In the present paper, we describe changes in risk perception among experts over time and evaluate outcomes of ACHD patients suffering from COVID-19 and how they differ overtime (first wave vs subsequent OCVID-19 waves).

## Methods

This prospective multicenter European registry was an initiative of the European Collaboration for Prospective Outcome Research in Congenital Heart disease (EPOCH, https://www.sacher-registry.com/epoch/).

### COVID-19 risk stratification survey

Back in April 2020, ACHD experts from different European centres and countries were invited to participate in a survey regarding risk stratification of ACHD patients with respect to anticipated COVID-19 outcome. Participants were asked whether they considered all ACHD patients to be at risk for COVID-19 related complications or not, and whether their standards for risk stratification were based on national or centre specific agreement, or personal judgment. Participants then had to select general and ACHD specific risk factors they considered as relevant for poor COVID-19 related outcome. Finally, participants estimated the risk of adverse COVID-19 outcomes in seven different common patient scenarios by scoring risks from 0 (no increased risk) to 100 (very high risk). Scores <25 were categorized as low risk, scores from 25 to 49 as low to moderate risk, scores from 50 to 74 as moderate to high risk and scores ≥75 as high risk. The detailed methodology of this survey has already been published [8]. Eleven months later (in March 2021), the same survey was again sent to the participants of the first round. Three additional items were then included. The results of valid matched pairs (survey 1 vs survey 2) were analysed. The new items were related to the value of registry data for risk estimation and to recommendations of the COVID-19 vaccine. The contain of the questionnaire was already [8] published and now included in the supplementary material (Table S1).

### Registry (COVID-19 tracker)

Twenty-six tertiary ACHD centres from nine European countries participated in this prospective, multicentre, cohort study. All ACHD patients who are tested positive for SARS-CoV-2 or with a strong clinical suspicion (clinical signs highly suggestive of COVID-19) presenting to or contacting one of the participating centres were included. Clinical observations were recorded and updated at regular time intervals until recovery or death and reported to the study coordinators at the University Hospital Inselspital in Bern, Switzerland. Data was initially collected weekly, later bi-weekly and monthly and were summarized in a report with all pertinent information related to demographics, clinical characteristics, and clinical outcomes of ACHD-patients with COVID-19, allowing incorporation of ‘real-time’ risk data into day-to-day clinical work at participating centres. (*Annex S1* of the supplementary material). The detailed methodology of this registry has already been published [9]. For purposes of this analysis, cases reported from March 27, 2020 (study begin) until May 6, 2021 (study termination) were included. A complicated disease course was defined as hospitalization for COVID-19 requiring ventilation and/or inotropic support, extracorporeal membrane oxygenation or death. Patient characteristics were stratified by COVID-19 wave (first vs. subsequent) being July 15, 2020 the cut-off date. The definition of listed comorbidities and residual heart defect-related problems was left to the discretion of the respondent, no pre-defined cut-offs were applied when defining advanced age and advanced renal or liver disease, heart failure or pulmonary or systemic arterial hypertension.

### Statistical considerations

SPSS software (version 26.0, SPSS Inc., Chicago, Illinois) and STATA 15 statistical software (Stata Corporation, College Station, TX, USA) were used for the analysis of the data. Distribution of continuous variables was assessed using skewness, kurtosis, and visual inspection of the histogram. For the comparison between surveys, results of nineteen valid matched pairs (survey 1 vs survey 2) were analysed. Data related to five surveys of the first round were excluded from the analysis because the corresponding ACHD experts did not answer to the second round of the survey. For the analysis of the COVID-19 Tracker, patients with missing data related to the date of COVID-19 diagnosis, as well as patients with missing outcome data were excluded. Continuous variables were presented as means (standard deviation [SD]) or medians (interquartile range [IQR]) and compared using paired *t*-test, or Wilcoxon, as appropriate, for the comparison of dependent samples (comparison of answers between the two surveys), and *t*-test or Mann–Whitney *U* test for the comparison of independent samples (stratified patient characteristics). Categorical variables were presented as counts (percentages) and compared using Chi-Square or McNemar tests, as appropriate. Predictors of the main variable of interest (complicated disease course) were analysed by univariable and multivariable logistic regression models calculated with cluster-robust standard errors. All statistically significant predictors of the outcome of interest in the univariable logistic regression mode were included into the multivariable model. Overall, the null hypothesis was rejected if p-value < 0.05.

The study complies with the 1975 Declaration of Helsinki and was approved by local research Ethics Committees according to local ethical policies and country specific regulations.

## Results

### Comparison between surveys

**Fig. 1:**
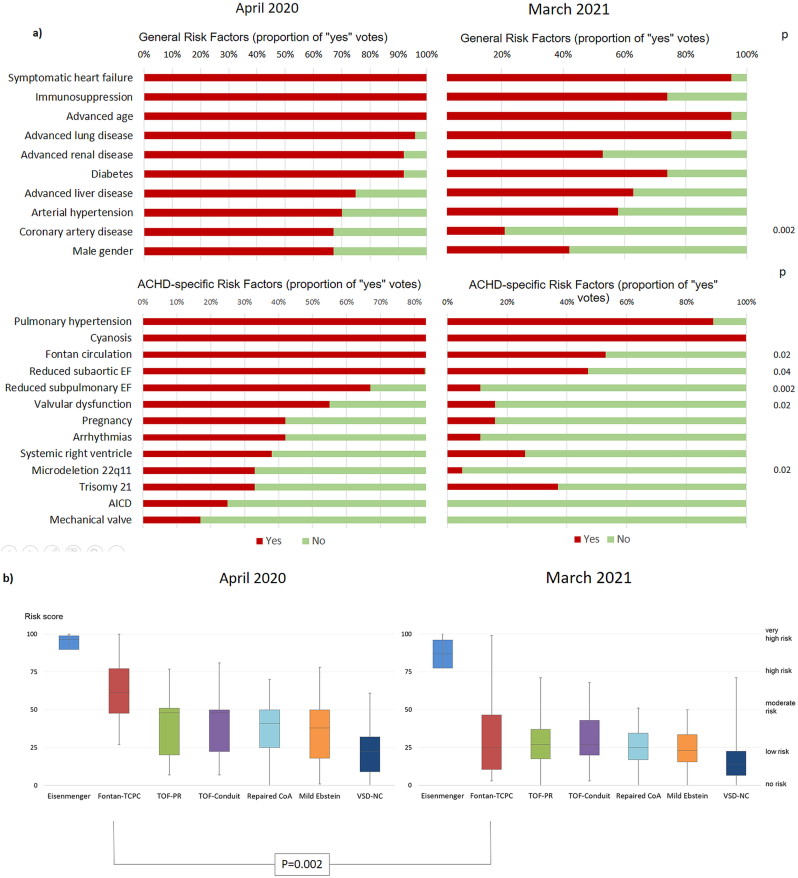
Comparison between surveys of answers related to general cardiovascular and ACHD-specific risk factors (a) and risk stratification among ACHD possible clinical scenarios (b). ACHD = adult congenital heart disease; AICD = automated implantable cardioverter defibrillator; EF = ejection fraction; Fontan-TCPC means univentricular physiology with total cavopulmonary connection and good hemodynamics; TOF-Conduit – repaired Tetralogy of Fallot with conduit implantation; TOF-PR – repaired Tetralogy of Fallot with residual severe pulmonary regurgitation; Repaired CoA – repaired aortic coarctation with mild residual hypertension; VSD-NC – repaired ventricular septal defect and mild non-compaction cardiomyopathy.

Overall, 43 surveys (24 from the first wave and 19 from the second one) were collected. A total of 19 valid matched pairs (survey 1 vs survey 2 for each participant) were analysed. A detailed comparison between surveys conducted in April 2020 and March 2021 is depicted in Table S2 and Fig. 1. In both surveys, only a minority of participating ACHD physicians considered all ACHD patients to be at risk in case of COVID-19. With the ongoing pandemic, registry data replaced national or working group consensus documents as primary source for risk stratification. At the time of the second survey, 95% of all participants relied on registry data for risk counselling. The use of other sources for this purpose did not importantly differ between surveys (Table S2). Fig. 1 depicts the perceived importance of general and ACHD specific risk factors for a complicated COVID-19 course. Among general risk factors, there was a trend for most variables to be considered of less importance in the second survey compared to the first one (Fig. 1 a). Similarly, among ACHD specific risk factors, all variables except from trisomy 21 and cyanosis were considered as less risky in the second survey (Fig. 1 a). This was especially true for the variables Fontan circulation, impaired (subaortic/subpulmonary) ventricular function, clinically relevant valvular heart disease and 22q11 microdeletion syndrome. Patients from all seven possible ACHD scenarios were considered to be at lower risk in the second survey when compared to the first one. Only the patient with Eisenmenger syndrome was still classified as a high-risk patient, while all other clinical scenarios where now classified as low-to-moderate or low-risk (Fig. 1 b). This was of particular relevance for the well doing Fontan patient with an extracardiac conduit, who was then considered to be at low risk (risk score of 62% vs 25%, p = 0.002).

### Analysis of the COVID-19 tracker

**Fig. 2:**
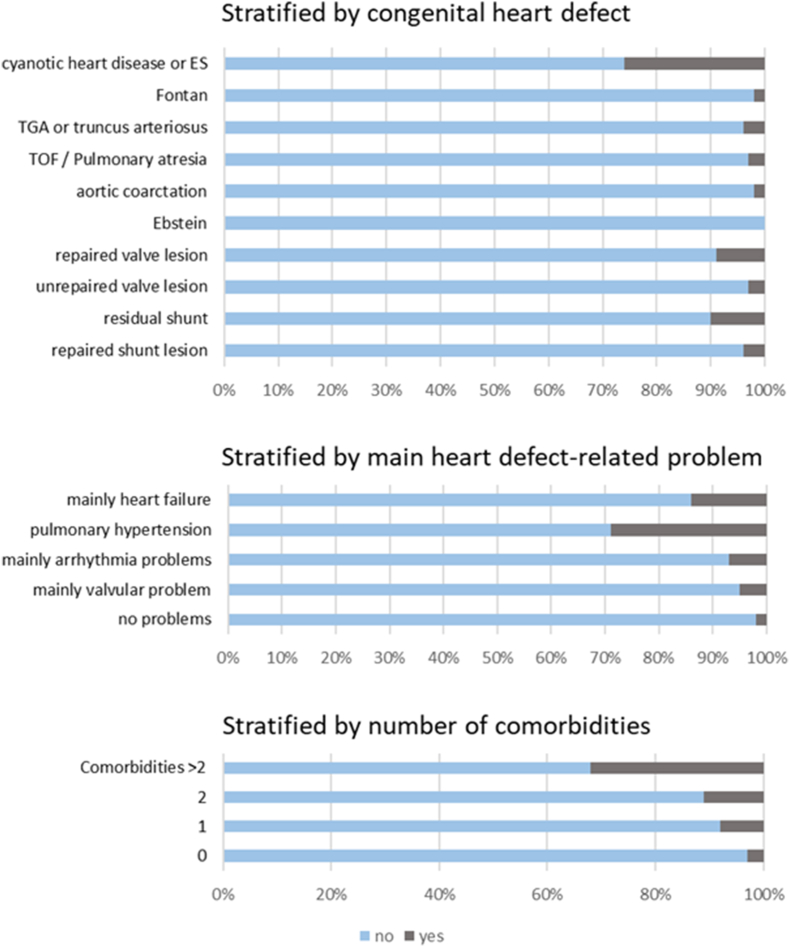
Proportion of cases with a complicated disease course. CHD = congenital heart defect; ES = Eisenmenger syndrome; TGA = transposition of the great arteries; TOF = tetralogy of Fallot.

From a total of 700 patients included into the registry, 638 individuals (91%, n = 168 for the first wave and n = 470 for the subsequent waves) with valid data on date of diagnosis and outcome were included. The median (IQR) age was 34 (26–44) years and 52% were women. The diagnosis of COVID-19 was confirmed by means of laboratory testing in 576 (90%) of the patients. Thirty-six percent of the patients had a cardiac defect of mild anatomical complexity, while defects of moderate and severe complexities were found in 35% and 29% of the participants, respectively. There were 36 (6%) cases with a complicated disease course, of whom 17 died (2.7% overall). The proportion of cases with a complicated disease course stratified by CHD and complexity, main residual heart defect-related problem and number of comorbidities is depicted in Fig. 2. Complicated cases were equally distributed among all levels of CHD complexity. Patients with heart failure and pulmonary hypertension, and those with ≥2 comorbidities seemed to be more prone to suffer a complicated disease course. The number of resolved, ongoing, and deceased cases over time is depicted in *Annex 1* of the supplementary material.

Patient characteristics stratified by COVID-19 wave are shown in Table 1. Patients from the first wave were older (median age of 37 vs. 33 years, p = 0.002), had more often ≥2 comorbidities (16% vs. 7%, p = 0.001) and a complicated disease course was more frequent when compared to those of the subsequent waves (9% vs. 5%, p = 0.03). Patients between waves did not differ with respect to gender, body mass index (BMI), cardiac defect complexity, residual heart defect-related problems and fatality rate.

**Table 1:** Patient characteristics stratified by coronavirus disease 2019-wave^a^.

*n* = *638 patients*	First wave (n = 168)	Subsequent waves (n = 470)	p
***Female gender, n (%)***	96 (57)	235 (50)	**0.11**
***Age in years***	37 (29–47)	33 (25–43)	**0.002**
***BMI, n (%)***			**0.83**
<25 kg/m^2^	105 (64)	304 (65)	
25–30 kg/m^2^	41 (24)	104 (22)	
>30 kg/m^2^	22 (13)	61 (13)	
***≥ 2 comorbidities, n (%)***	27 (16)	34 (7)	**0.001**
***Cardiac defect complexity, n (%)***			**0.23**
Mild	63 (38)	167 (36)	
Moderate	65 (39)	159 (34)	
Severe	40 (24)	144 (31)	
***Residual defect-related problems, n (%)***			**0.25**
no problems	64 (38)	225 (48)	
mainly valvular problem	61 (36)	147 (31)	
mainly heart failure	15 (9)	34 (7)	
mainly arrhythmia problems	20 (12)	41 (9)	
pulmonary hypertension	8 (5)	23 (5)	
**Complicated disease course, n (%)**	15 (9)	21 (5)	**0.03**
**Deaths, n (%)**	7 (4)	10 (2)	**0.18**

Data are median (interquartile range) or number (percentage). BMI = body mass index (in kg/m2).

a July 15, 2020 was the cut-off date.

In univariable analysis, male sex, increasing age, a BMI >25 kg/m2, having ≥2 comorbidities, suffering from a cyanotic heart disease (including Eisenmenger syndrome) or having suffered from COVID-19 in the first wave vs. subsequent waves were predictive of a complicated disease course (Table 2). In the multivariable analysis all the above-mentioned variables remained independently associated with the outcome of interest.

**Table 2:** Predictors for complicated coronavirus disease 2019-course.

Predictor	Odds ratio	95% Confidence interval	p
***Univariable logistic regression***			
*Sex (female)*	0.65	(0.52–0.80)	<0.001
*Age (per 5 years)*	1.36	(1.25–1.48)	<0.001
*Cardiac defect complexity (severe)*	1.57	(0.76–3.24)	0.18
*BMI >*25 kg/m^2^	3.28	(1.94–5.54)	<0.001
*Cyanotic heart disease or ES*	7.38	(3.69–14.77)	<0.001
*≥ 2 comorbidities*	5.59	(4.22–7.40)	<0.001
*COVID-19 wave (first)*	2.10	(1.58–2.78)	<0.001
***Multivariable logistic regression***			
*Sex (female)*	0.49	(0.37–0.66)	<0.001
*Age (per 5 years)*	1.22	(1.04–1.43)	0.02
*Cyanotic heart disease or ES*	8.23	(3.35–20.21)	<0.001
*BMI >*25 kg/m^2^	2.58	(1.19–5.61)	0.02
*≥ 2 comorbidities*	2.41	(1.46–3.98)	0.001
*COVID-19 wave (first)*	1.96	(1.36–2.90)	<0.001

All statistically significant predictors of the outcome of interest in the univariable logistic regression mode were included into the multivariable model.

Total number of events: 36. BMI = body mass index; COVID-19 = coronavirus disease 2019; ES = Eisenmenger syndrome.

A comprehensive description of the clinical course and the patient characteristics of the fatal cases is presented in Table S3 of the supplementary material. The above mentioned characteristics of the first five patients in Table S3 have been already published [9], while information related to the last twelve patients has newly been added.

The proportion of deaths among the reported cases was 2.7% (17/638). This number was similar to the proportion of fatal cases of the general population among the participating countries (2.3%) [10]. See Table S4 of the supplementary material for details. Comorbidities were less prevalent among ACHD patients of our cohort when compared to reference cohorts of hospitalized patients due to COVID-19 [11,12]. Only immunosuppression among hospitalized patients and renal failure among intensive care unit patients were less prevalent in the reference cohort than in our study population (Table S5). As expected, ACHD patients were younger than those from the reference population, even among hospitalized patients and among those with a complicated COVID-19 course (median age 47 vs. 63 years and 49 vs. 63 years, respectively).

## Discussion

### Risk stratification of ACHD patients during the current pandemic

This study describes changes in risk stratification of ACHD patients during the current pandemic by comparing the results of two surveys sent to experts in the field of ACHD at two different time-points along the pandemic: at the beginning and shortly after the first outcome data on ACHD patients suffering from COVID-19 were available. When assessing the importance of general and ACHD specific risk factors for a complicated disease course in case of COVID-19 among our patients, the overall risk perception was lower in the second survey when compared to the first one. This was true even for risk factors related to physiological stage (such as ventricular function and valvular heart disease), which has recently been associated with poor outcomes among ACHD patients suffering from COVID-19 [13]. From all seven possible ACHD clinical scenarios, all patients were considered to be at lower risk in the second survey. This was particularly noticeable among patients with Fontan circulation, as several studies published after the first survey failed to demonstrate an increased risk among otherwise well doing patients with a Fontan physiology in case of COVID-19 [9,13,14]. In general, risk stratification among experts tended to be more homogeneous in the second round of the survey.

The fact that perceived risk for patients among ACHD experts was greater at the beginning of the pandemic is reflected by position papers and recommendations based on expert opinions that were published in the absence of outcome data and corresponds to the very human reaction of being rather cautious when confronted with an unknown hazard [[15], [16], [17]]. The fact that the vast majority (95%) of the experts based the COVID-19 risk estimation of their patients on information derived from registries (such as the current study) deserves special mention and speaks for a key role of these tools in global health challenges such as the current pandemic.

### The COVID-19 tracker

In our study, main independent predictors for a complicated disease course were male sex, increasing age, being overweight or obese, having ≥2 comorbidities, suffering from a cyanotic heart disease (including Eisenmenger syndrome) or having suffered from COVID-19 in the first wave. Our results are in line with previously reported outcome data among ACHD patients suffering from COVID-19 [13,18]. Compared to our previous report on the matter, female gender was now a protective characteristic for a complicated disease course [18]. This is consistent with the observations found by Broberg et al., and with those seen in reports from the general population with male sex associated with a poor outcome in case of COVID-19 [19,20]. This discrepancy between our previous and current reports may be explained due to the limited number of cases and outcomes in our first analysis. Furthermore, the results of our study for the first time revealed that ACHD patients infected with COVID-19 during the first wave were older and had more comorbidities than those from subsequent waves. This may reflect how older patients and those with comorbidities were more prone to comply with primary prophylactic measures implemented during and after the first wave (such as social distancing and lockdown-related measures). One might argue that these facts (the older age and the higher comorbidity burden) could be the sole explanation for the higher proportion of cases with a complicated disease course (15 (9%) vs. 21 (5%), p = 0.03) observed in the first wave when compared to the following ones (mortality was similar between waves [7 (4%) vs. 10 (2%), p = 0.18]). However, the fact that COVID-19 wave (first vs. subsequent) remained an independent predictor for complicated disease course in multivariable analysis points towards the important role of improved medical care (including vaccination) for patients affected by COVID-19 along the progression of the COVID-19 pandemic.

Interestingly, the complexity of the congenital heart defect per se did not seem to correlate with the outcome in case of COVID-19. As shown in Fig. 2, the proportion of cases with a complicated disease course was equally distributed among patients with severe, moderate and mild anatomical complexity. Having a cardiac defect of great complexity was not a predictor for a complicated disease course in case of COVID-19. These observations are also consistent with our previous report, and with the one from Broberg et al. and are unexpected. Cardiac anatomy was considered to play a key role in the classification of ACHD patients into low and high risk proposed in the position paper from the ESC working group of adult congenital heart disease and the International Society for Adult Congenital Heart Disease [15]. Because of the above mentioned, when considering risk stratification of ACHD patients, attention should be rather focus on their comorbidity burden (i.e. Systemic arterial hypertension, cardiovascular disease, chronic obstructive pulmonary disease) and their physiological stage, often determined by anatomic and hemodynamic sequelae derived from previous surgeries and interventions (i. e. overt heart failure, pulmonary hypertension, arrhythmia) [13,21].

When comparing outcomes of ACHD patients suffering from COVID-19 to those encountered in the general population, the proportion of deaths was comparable (2.7% vs. 2.3%) [10]. This might be surprising if we consider the overall lesser comorbidity burden and the younger age of ACHD patients in our cohort when compared to reference cohorts of hospitalized patients due to COVID-19 [11,12]. This again indicates the important role of physiological stage and hemodynamics among ACHD patients.

## Limitations

Considering the small sample size of both surveys, the statistical analysis for the comparison of the nineteen matched pars might have been underpowered. As, we only included patients diagnosed with COVID-19 or strong clinical suspicion presenting to or contacting one of the participating centres, we may have missed some cases, especially those with a mild disease course. This is particularly true for the first weeks of the pandemic, when testing strategies were still being developed. Therefore, we cannot make any valid estimations on the prevalence of COVID-19 among the ACHD population. Furthermore, because no specific definitions or predefined cut-offs were used when regarding variables such as advanced renal or lung disease, heart failure, systemic and/or pulmonary arterial hypertension, and advanced age, these variables were excluded from the main analysis and their potential predictive value for the outcome of interest could not be assessed by means of logistic regression models. However, the higher proportion of cases with a complicated disease course among those classified by the reporting physician as having pulmonary arterial hypertension or overt heart failure is consistent with the data from Broberg et al. and the overall idea that physiological stage and comorbidities play a major role in the risk of this patients [13]. Finally, as data related to vaccination status among participant is missing, no conclusion can be made regarding the potential effect of the SARS-CoV-2 vaccine on our results.

## Conclusion

Risk stratification of ACHD patients in the current pandemic was challenging and dynamic over time. Our registry and its ‘real-time’ outcome reports, significantly helped improving risk stratification along the time-course of the pandemic. Risk factors for poor outcome in case of COVID-19 seen in the general population are also determinants of outcome among ACHD patients. While patients with cyanotic heart diseases were at risk for poor outcome in case of COVID-19, the anatomical complexity of the congenital heart disease per se did not seem to be related to morbidity and/or mortality in case of COVID-19. Special attention should be paid to physiological stage of our patients (i. e. a patient with a repaired atrial septum defect and severe residual pulmonary arterial hypertension). The experience gained during the first wave of the pandemic helped improving the prognosis of patients of subsequent ones. International collaborations play a major role when aiming to deliver evidence base recommendation for ACHD patients.

## Contributorship statement

FJRR, DT, MG, MS and JB contributed to drafting of the manuscript, the conception of the research, critical revision of the manuscript for important intellectual content and supervision. All other authors contributed to the patient recruitment, data collection and the critical revision of the manuscript for important intellectual content and supervision. The corresponding author attests that all listed authors meet authorship criteria and that no others meeting the criteria have been omitted.

## Funding

EPOCH is funded by internal grants without support from the pharmaceutical industry.

## Declaration of competing interest

The authors declare the following financial interests/personal relationships which may be considered as potential competing interests:Professor Werner Budts reports financial support was provided by Abbott and Occlutech. Following co-authors: Dr. Pastora Gallego, Dr. Magalie Ladouceur, Dr. Massimo Chessa, Professor Werner Budts and Prof. Helmut Baumgartner are Associate Editors and, therefore, members of the Editorial Board of the International Journal of Cardiology Congenital Heart Disease.

## References

[1] Zisman L.S., Keller R.S., Weaver B., Lin Q., Speth R., Bristow M.R. (2003). Increased angiotensin-(1-7)-forming activity in failing human heart ventricles: evidence for upregulation of the angiotensin-converting enzyme Homologue ACE2. Circulation.

[2] Guzik T.J., Mohiddin S.A., Dimarco A., Patel V., Savvatis K., Marelli-Berg F.M. (2020). COVID-19 and the cardiovascular system: implications for risk assessment, diagnosis, and treatment options. Cardiovasc Res.

[3] Shi S., Qin M., Shen B., Cai Y., Liu T., Yang F. (2020). Association of cardiac injury with mortality in hospitalized patients with COVID-19 in wuhan, China. JAMA cardiology.

[4] Hoffmann M., Kleine-Weber H., Schroeder S., Krüger N., Herrler T., Erichsen S. (2020). SARS-CoV-2 cell entry depends on ACE2 and TMPRSS2 and is blocked by a clinically proven protease inhibitor. Cell.

[5] Wu C., Chen X., Cai Y., Xia J., Zhou X., Xu S. (2020). Risk factors associated with acute respiratory distress syndrome and death in patients with coronavirus disease 2019 pneumonia in wuhan, China. JAMA Intern Med.

[6] Wu Z., McGoogan J.M. (2020). Characteristics of and important lessons from the coronavirus disease 2019 (COVID-19) outbreak in China: summary of a report of 72 314 cases from the Chinese center for disease control and prevention. JAMA.

[7] Zhou F., Yu T., Du R., Fan G., Liu Y., Liu Z. (2020). Clinical course and risk factors for mortality of adult inpatients with COVID-19 in Wuhan, China: a retrospective cohort study. Lancet.

[8] Ruperti-Repilado F.J., Tobler D., Greutmann M., Bouchardy J., Ladouceur M., Dos-Subira L. (2021). Risk stratification of adults with congenital heart disease during the COVID-19 pandemic: insights from a multinational survey among European experts. Open Heart.

[9] Schwerzmann M., Ruperti-Repilado F.J., Baumgartner H., Bouma B., Bouchardy J., Budts W. (2021). Clinical outcome of COVID-19 in patients with adult congenital heart disease. Heart.

[10] https://coronavirus.jhu.edu/map.html, last visited on June, 4th at 11:00 pm (GMT).

[11] Grasselli G., Greco M., Zanella A., Albano G., Antonelli M., Bellani G. (2020). Risk factors associated with mortality among patients with COVID-19 in intensive care units in lombardy, Italy. JAMA Intern Med.

[12] Richardson S., Hirsch J.S., Narasimhan M., Crawford J.M., McGinn T., Davidson K.W. (2020). Presenting characteristics, comorbidities, and outcomes among 5700 patients hospitalized with COVID-19 in the New York city area. JAMA.

[13] Broberg C.S., Kovacs A.H., Sadeghi S., Rosenbaum M.S., Lewis M.J., Carazo M.R. (2021). COVID-19 in adults with congenital heart disease. J Am Coll Cardiol.

[14] Fusco F., Scognamiglio G., Merola A., Palma M., Correra A., Borrelli N. (2021). Coronavirus disease 2019 in patients with Fontan circulation. Int J Cardiol Congenit Heart Dis.

[15] Diller G.P., Gatzoulis M.A., Broberg C.S., Aboulhosn J., Brida M., Schwerzmann M. (2020). Coronavirus disease 2019 in adults with congenital heart disease: a position paper from the ESC working group of adult congenital heart disease, and the International Society for Adult Congenital Heart Disease. Eur Heart J.

[16] Gallego P., Ruperti-Repilado F.J., Schwerzmann M. (2020). Adults with congenital heart disease during the coronavirus disease 2019 (COVID-19) pandemic: are they at risk?. Rev Esp Cardiol.

[17] Radke R.M., Frenzel T., Baumgartner H., Diller G.P. (2020). Adult congenital heart disease and the COVID-19 pandemic. Heart.

[18] Schwerzmann M., Ruperti-Repilado F.J., Baumgartner H., Bouma B., Bouchardy J., Budts W. (2021).

[19] Suleyman G., Fadel R.A., Malette K.M., Hammond C., Abdulla H., Entz A. (2020). Clinical characteristics and morbidity associated with coronavirus disease 2019 in a series of patients in metropolitan detroit. JAMA Netw Open.

[20] Nguyen N.T., Chinn J., De Ferrante M., Kirby K.A., Hohmann S.F., Amin A. (2021). Male gender is a predictor of higher mortality in hospitalized adults with COVID-19. PLoS One.

[21] Yuan S., Oechslin E. (2021). Perception is not reality when risk stratifying adults with congenital heart disease for COVID-19. Open Heart.

