# Supplementary material for: The coronavirus disease pandemic among adult congenital heart disease patients and the lessons learnt – results of a prospective multicenter european registry
Source: Int J Cardiol Congenit Heart Dis. 2022 Nov 21;11:100428. doi: 10.1016/j.ijcchd.2022.100428 (PMC9678209; doi:10.1016/j.ijcchd.2022.100428)
Supplement: Multimedia component 1 [file mmc1.docx]

**Supplementary Material**

**Table S1. Questionnaire of the first round**

| Question | Answer | |
| --- | --- | --- |
| *1. Please, indicate your name* |  | |
| *2. Do you consider all ACHD patients at risk for COVID-19 related complications* | - Yes  - No  - Unsure | |
| *3. How do you estimate COVID risk in your patients (multiple answers are possible)* | - based on national / working group consensus  - based on center specific agreement  - based on personal judgment | - I don’t stratify ACHD patients into different COVID-19 risk categories  - I don’t know |
| *4. What do you consider as general risk factor(s) for an adverse outcome in COVID-19 (multiple answers are possible)* | - diabetes  - advanced renal disease  - arterial hypertension  - male gender  - immunosuppression  - coronary artery disease | - symptomatic heart failure  - advanced liver disease  - advanced age  - advanced lung disease  - any other condition not mentioned before: |
| *5. What do you consider as ACHD-specific risk factor(s) for an adverse outcome in COVID-19 (multiple answers are possible)* | - pregnancy  - impaired subpulmonary ventricular function  - trisomy 21  - mechanical heart valve(s)  - cyanotic heart disease (SpO2 <90%)  - 22q11 microdeletion  - moderate or severe valvular heart disease | - symptomatic arrhythmia  - pulmonary arterial hypertension  - systemic right ventricle (per se)  - Fontan physiology  - impaired subaortic ventricular function  - ICD carrier  - any other condition not mentioned before |
| *6. What do you consider as most important risk factor for an adverse outcome in COVID-19 (please rate the following options from 1 to 4, with 1=most important)* | - complexity of heart defect  - age  - gender  - co-morbidities | |
| *7. Do you consider this patient at risk for COVID-19 related complications?*  *Please rate from 0% (no risk) to 100% (very high risk)* | 25-year-old male Fontan patient with an extracardiac TCPC-conduit and good hemodynamics, no arrhythmia. | |
| *8. Do you consider this patient at risk for COVID-19 related complications?*  *Please rate from 0% (no risk) to 100% (very high risk)* | 35-year-old male with repaired coarctation of the aorta, a mildly stenotic bicuspid aortic valve and persistent proximal hypertension, well controlled with ACE-inhibitors | |
| *9. Do you consider this patient at risk for COVID-19 related complications?*  *Please rate from 0% (no risk) to 100% (very high risk)* | 35-year-old female with Ebstein anomaly with mild to moderate tricuspid valve regurgitation and patent foramen ovale | |
| *10. Do you consider this patient at risk for COVID-19 related complications?*  *Please rate from 0% (no risk) to 100% (very high risk)* | 40-year-old male with repaired Tetralogy of Fallot, a mildly stenotic RV-PA conduit and a right ventricle with a RV-EDVi of 110 ml/m2 and an RV-EF of 38%. | |
| *11. Do you consider this patient at risk for COVID-19 related complications?*  *Please rate from 0% (no risk) to 100% (very high risk)* | 25-year old male with patch closure of a ventricular septal defect and left-ventricular non-compaction with an LV-EF of 50%. | |
| *12. Do you consider this patient at risk for COVID-19 related complications?*  *Please rate from 0% (no risk) to 100% (very high risk)* | 45-year-old male with repaired Tetralogy of Fallot and moderate to severe pulmonary regurgitation, a right ventricle with a RV-EDVi of 140 ml/m2 and an RV-EF of 49%. | |
| *13. Do you consider this patient at risk for COVID-19 related complications?*  *Please rate from 0% (no risk) to 100% (very high risk)* | 45-year-old female with trisomy 21 and Eisenmenger physiology due to an non-restrictive ventricular septal defect (SpO2 78%) and preserved biventricular function) | |

ACE means angiotensin-converting-enzyme; ACHD, congenital heart disease; COVID-19, coronavirus disease 2019; ICD, implantable cardioverter defibrillator; SpO2, peripheral capillary oxygen saturation; LV-EF, left ventricle ejection fraction; TCPC, total cavopulmonary connection; RV-EDVi, right ventricle end-diastolic volume index; RV-EF, right ventricle ejection fraction and RV-PA, right ventricle to pulmonary artery.

No pre-defined cut-offs were applied when defining advanced age and advanced renal / liver disease

**Table S2. Comparison of answers between the two surveys***

| **Question** | **April 2020** | **March 2021** |
| --- | --- | --- |
|  | **n=24** | **n=19** |
| **All ACHD patients at risk in case of COVID-19? (yes)** | 4 (17) | 3 (13) |
| **How do you estimate COVID-19 risk in your patients?** |  |  |
| *national / working group consensus* | 18 (75) | 11 (58) |
| *center-specific agreement* | 7 (29) | 6 (32) |
| *personal judgment* | 14 (58) | 10 (52) |
| *based on registry data* | - | 18 (95) |
| **Risk factors regarding adverse outcomes in COVID (1 = most important, 4 = least important)** |  |  |
| *complexity of the heart defect* | 2.17 | 3.47 |
| *age* | 2.21 | 1.68 |
| *co-morbidities* | 2.13 | 2.26 |
| *gender* | 3.5 | 3.58 |
| **COVID-19 vaccination to all ACHD patients recommended?** | - | 19 (100) |
| **Usefulness of the reports to daily clinical practice? (0: not useful – 100: very useful)***^+^* | - | 79 |

ACHD= adult congenital heart disease, COVID-19= coronavirus disease 2019

Data are reported as numbers (percentage) and means, as appropriate.

^*^ The comparison of questions related to acquired cardiovascular and ACHD-specific risk factors, as well as the comparison of risk scores among the different ACHD possible clinical scenarios are graphically depicted in *Figue 2*

^+^ The question/answer was not available in the first questionnaire

**Table S3 Comprehensive description of fatal outcomes**

| **Age** | **Sex** | **Congenital heart defect** | **Clinical course** | **Proposed causality of the death** |
| --- | --- | --- | --- | --- |
| 40-50 | Male | Repaired Tetralogy of Fallot | Pre-existing severe biventricular dysfunction and progressive heart failure (had implanted CRT-D), cardiac-related liver cirrhosis and right lung hypoplasia due to an occluded right pulmonary artery. Decision regarding cardiac and liver-transplant was pending.  Admitted with ARDS. Due to comorbidities the patient was not considered a candidate for extensive cardio-respiratory support. He died at day 3 after hospital admission. | Death due to SARS-CoV-2 infection (ARDS related to COVID-19)  Underlying CHD may have contributed to fatal outcome.  Co-morbidities/heart defect-related problems: 3 (heart failure, liver and lung disease). |
| >60 | Male | Repaired pulmonary valve stenosis | Mild pulmonary regurgitation and acquired cardiovascular disease (coronary artery disease, s/p ischemic stroke, abdominal aneurysm, atrial fibrillation) and COPD. NYHA class II prior to COVID-19.  Admitted with bilateral pneumonia leading to ARDS requiring intubation at the day of admission. Renal failure occurred 3 days after presentation. He died at day 11 after admission with multiorgan failure. | Death due to SARS-CoV-2 infection (ARDS related to COVID-19)  Underlying CHD unlikely to contribute to fatal outcome.  Co-morbidities/heart defect-related problems: 3 (previous stroke, coronary artery disease, lung disease) |
| 40-50 | Female | Bicuspid aortic valve with severe aortic stenosis | Presentation with decompensated heart failure due to severe aortic stenosis, requiring urgent surgical aortic valve replacement. Complicated postoperative course with cardiogenic shock requiring veno-arterial ECMO.  Developed ARDS on first postoperative day and tested positive for SARS-CoV-2. She died 7 days after surgery. | Death with SARS-CoV-2 infection; (Postoperative death due to heart failure)  Underlying CHD was the main reason for the fatal outcome.  Co-morbidities/heart defect-related problems: 1 (heart failure) |
| 50-60 | Female | Eisenmenger syndrome with unrepaired complete atrioventricular septal defect | Severe pulmonary hypertension, heart failure and moderate leucopenia. Presentation at the emergency department with bilateral pneumonia and ARDS.  Due to her functional status (NYHA class III) prior to COVID-19 and personal preferences, she was transferred to a palliative care center. She died at day 32 after initial hospital admission. | Death due to SARS-CoV-2 infection (ARDS related to COVID-19)  Underlying CHD contributed to fatal outcome.  Co-morbidities/heart defect-related problems: 3 (trisomy 21, heart failure, pulmonary hypertension) |
| 40-50 | Female | Eisenmenger syndrome with unrepaired complete atrioventricular septal defect | Severe pulmonary hypertension, heart failure and severely reduce renal function. Presentation at the emergency department with ARDS.  Due to her functional status (NYHA class IV) prior to COVID-19 and personal preferences, she was discharged home. She died at home 22 days after initial hospital presentation. | Death due to SARS-CoV-2 infection (ARDS related to COVID-19)  Underlying CHD contributed to fatal outcome.  Co-morbidities/heart defect-related problems: 4 (trisomy 21, heart failure, pulmonary hypertension, kidney failure) |
| 50-60 | Male | Partial anomalous pulmonary venous drainage  Persistent foramen ovale (PFO) with severe right-to-left shunt | History of type 2 diabetes mellitus and esophageal cancer. Incidental diagnosis of partial anomalous pulmonary venous drainage during the diagnostic cancer workup. Normal right ventricular dimensions, no evidence of pulmonary hypertension. Hospital admission for elective esophagectomy. Recurrent postoperative hypoxemia requiring re-intubation. Diagnosed with COVID-19 on postoperative day 4. Subsequently severe ARDS with hemodynamic instability, severe pulmonary hypertension and multiple secondary infectious complications. Diagnosis of a patent foramen ovale with severe right-to-left shunting on postoperative day 23. Emergent veno-arterial ECMO on postoperative day 26 and percutaneous PFO closure on postoperative day 27. Weaning from ECMO 7 days after PFO-closure. The patient recovered, was discharged and died during inpatient rehabilitation. | Death still unclear  Admission for noncardiac surgery  Patent foramen ovale was a contributor to complicated disease course. Partial anomalous pulmonary venous return likely not substantially contributing to disease course.  Co-morbidities/heart defect-related problems: 2 (diabetes, cancer) |
| 40-50 | Male | Eisenmenger due to unrepaired VSD | Patient institutionalized at the time of COVID-19 diagnosis. Initially treated at his institution with supplemental oxygen and subcutaneous morphine. Referral to his regional hospital of reference due to impaired consciousness secondary to morphine intoxication in the context of worsened renal failure. Progressive respiratory failure over time, probably due to bacterial pulmonary superinfection. Patient/family did not desired invasive mechanical ventilation. Therefore, a palliative approach was stablished. The patient deceased shortly thereafter. | Death due to SARS-CoV-2 infection  Underlying CHD contributed to death  Co-morbidities/heart defect-related problems: 2 (Trisomy 21, renal failure stage IIIb) |
| 40-50 | Male | Repaired AVSD with mechanical mitral valve prosthesis.  Thranscatheter aortic coarctaction repair before AVSD surgery | No significant complications before admission. Admitted to hospital because of endocarditis of mitral valve prosthesis complicated with an ischemic stroke. Positive PCR for Sars-Cov2 on admission screening. Intubated and on inotropic drugs for almost 2 months due to multisystem organ failure and ARDS. | Death with SARS-CoV-2 infection; (Multisystem organ failure and ARDS).  Underlying complication of CHD was the main reason for the fatal outcome.  Co-morbidities/heart defect-related problems: 1 (immunocompromised: IgG4 related disease on corticosteroids) |
| 50-60 | Male | Repaired ventricular septal defect.  Bio-aortic valve replacement (3x) due to endocarditis. Residual moderate paravalvular aortic valve regurgitation, aortic root aneurysm. | History of recurrent infective endocarditis (3x). Arterial hypertension. Institutionalized due to his psychiatric history (bipolar disorder). Recurrent episodes of sustained ventricular tachycardia. ICD implantation discussed 1 year ago with health care proxy but finally not performed due to fear of inappropriate shocks. Recurrent hospitalisations for decompensated heart failure with preserved ejection fraction. The patient tested positive for SARS-CoV-2 infection at his institution during a general outbreak. He presented mild symptoms (fever) and was already recovering 4 days after the diagnosis. No signs or symptoms of decompensated heart failure during convalescence. Sudden death during the night. | Death with SARS-CoV-2 infection; cause unclear; probably related to arrhythmia.  Underlying CHD and acquired severe aortic regurgitation were probably the main reasons for the fatal outcome.  Co-morbidities/heart defect-related problems: 2 (arterial hypertension, heart failure) |
| 40-50 | Female | Severe mitral valve regurgitation due to parachute mitral valve | Patient lost to follow-up since 2016. History of congestive heart failure secondary to severe mitral valve regurgitation due to parachute mitral valve. She was completely dependent for activities of daily living due to cerebral palsy with severe psychomotor retardation and epilepsy. No radiological signs of pneumonia at diagnosis. She stayed at home during convalescence. She died two months and eight days after COVID-19 diagnosis due to progressive deterioration of her overall medical condition (dehydration and anorexia). | Death after SARS-CoV-2 infection; cause unclear. Respiratory failure due to worsening of chronic heart failure in the context of progressive deterioration of her overall medical condition was postulated.  Underlying CHD likely to contribute to fatal outcome.  Co-morbidities/heart defect-related problems: 2 (cerebral palsy, heart failure) |
| 20-30 | Female | Repaired AVSD with mechanical mitral valve prosthesis | Anticoagulation with subcutaneous low-molecular-weight heparin due to mechanical mitral valve prosthesis. Otherwise healthy woman at 29 weeks of pregnancy. Hospital admission due to respiratory failure 18 days after diagnosis of COVID-19, for which she was treated with steroids at home until the day before admission. At admission, a transthoracic echocardiography showed normal biventricular function and pronounced D-shaping with concomitant obstruction of the LVOT. Rapid transfer to the ICU due to refractory respiratory insufficiency and hypotension, where invasive mechanical ventilation was started. Legionella urinary antigen testing was twice positive. Broad-spectrum antimicrobial treatment was started. A transesophageal echocardiography ruled-out a thrombosis of the mechanical mitral valve prosthesis. An emergency caesarean section was successfully performed. During surgery, no relevant blood loss occurred. Rapid deterioration of the respiratory and hemodynamic situation back at the ICU with refractory shock and multiorgan failure. The patient died the day after admission. | Death due to SARS-CoV-2 infection (ARDS related to COVID-19-related bacterial superinfection)  Underlying CHD may have contributed to fatal outcome.  Co-morbidities/heart defect-related problems: 0 |
| 0 | Female | Cor triatriatum sinistrum, bicuspid aortic valve and VSD  VCD closure at 5 y.o  Intra atrial membrane resection at 43 y.o | History of severe asthma, Type 2 diabetes mellitus and renal dysfunction (GFR= 37 mL/min/1.73m2). Transferred for cardiovascular rehabilitation after hospital admission for heart failure and atrial arrhythmia. Admitted to the intensive care four weeks after discharge due respiratory failure in the context of bilateral pneumonia related to COVID-19. She died 10 days after admission in the ICU due to multisystem organ failure and ARDS | Death due to SARS-CoV-2 infection (ARDS related to COVID-19)  Underlying CHD may have contributed to fatal outcome.  Co-morbidities/heart defect-related problems: 7 (heart failure, atrial arrhythmia, renal dysfunction, diabetes, hypertension, asthma, obstructive sleep apnea). |
| >60 | Male | ASD II diagnosed late in life due to pulmonary embolism.  Closure due to missing hemodynamic significance and elevated arterial pulmonary pressure/restrictive RV not performed. | Presentation with worsening dyspnea. COVID-19 was diagnosed upon admission. A treatment with Remdesivir and Dexamethason were started. Referral to the intensive care unit for non-invasive ventilation due to progressive hypoxemia. Rapid deterioration with respiratory insufficiency and ARDS. Because of his general condition and multiple co-morbidities, invasive mechanical ventilation was discard. The patient died shortly thereafter. | Death due to SARS-CoV-2 infection (ARDS related to COVID-19).  Underlying CHD and specially age co-morbidities (overall general condition) contributed to the fatal outcome.  Co-morbidities/heart defect-related problems: 5 (diabetes, cerebrovascular disease, multiple thromboembolic episode, chronic hypoxemia, hypertension). |
| 50-60 | Male | Unrepaired L-TGA, complete heart block | Patient with history of systemic right ventricular dysfunction, PM for complete heart block and permanent atrial arrhythmia. Diagnosis of pre and post-capillary arterial hypertension during heart transplantation work-up (PVR= 8.82 Wood units) contraindicating heart transplantation. Hospitalized for cardiogenic shock two months after CRT-D implantation requiring cardio-pulmonary support. COVID-19 during hospital stay with rapid hemodynamic deterioration. A palliative approach was stablished. The patient deceased shortly thereafter. | Death due to cardiogenic shock related to advance heart failure and COVID-19 pneumonia.  Underlying CHD contributed to the fatal outcome  Co-morbidities/heart defect-related problems: 1 (heart failure) |
| 30-40 | Female | Eisenmenger syndrom due to unrepaired VSD | Presentation with worsening dyspnea, severe cyanosis and hemoptysis. COVID-19 was diagnosed upon admission. Due to her functional status, the patient was included in a palliative care program. She deceased 2 days after admission. | Death due to SARS-CoV-2 infection (ARDS related to COVID-19)  Underlying CHD have contributed to the fatal outcome.  Co-morbidities/heart defect-related problems: 2 (Trisomy 21, pulmonary hypertension) |
| >60 | Male | Repaired Tetralogy of Fallot  RV-PA conduit implantation in 2005 and replacement in 2021 | Irregular follow-up with history of severely reduce right ventricular systolic function due to severe RV-PA conduit stenosis and right pulmonary artery stenosis. Admission for elective replacement of the RV-PA conduit and patch augmentation of the right pulmonary artery. Prolonged postoperative convalescence due to right heart failure. Nosocomial COVID-19 during hospital stay with rapid development of severe bilateral pneumonia with rapid progression to ARDS, bacterial superinfection, multisystem failure and death. | Death due to SARS-CoV-2 infection (ARDS related to COVID-19-related bacterial superinfection)  Underlying CHD may have contributed to fatal outcome.  Co-morbidities/ heart defect-related problems: 1 (heart failure) |
| 50-60 | Male | Repaired valve lesion | Further information related to the fatal outcomes of this patient was not provided |  |

ARDS: acute respiratory distress syndrome; CHD: congenital heart defect; COPD: chronic obstructive pulmonary disease; COVID-19: coronavirus disease 2019; CRT-D: cardiac resynchronization therapy defibrillator; ECMO: extracorporeal membrane oxygenation; NYHA: New York Heart Association; PM: pacemaker; RV-PA: right ventricular to pulmonary artery; SARS-CoV-2: severe acute respiratory syndrome coronavirus 2.

| **Country** | **Study population** | **General population^*^** |
| --- | --- | --- |
| *Austria* | 0 / 4 (0%) | 10’700 / 650’353 (1.6%) |
| *Belgium* | 1 / 61 (1.7%) | 25’168 / 1’083’478 (2.3%) |
| *Denmark* | 1 / 20 (5%) | 2’478 / 249.020 (1%) |
| *France* | 3 / 39 (7.7%) | 111’175 / 5’832’721 (1.9%) |
| *Germany^+^* | - / 1 | 90’833 / 3’734’955 (2.4%) |
| *Italy* | 0 / 2 (0) | 127’500 / 4’258’456 (3%) |
| *Netherlands, the* | 2 / 80 (2.5%) | 18’028 / 1’712'083 (1.1%) |
| *Spain* | 7 / 283 (2.5%) | 80'789 / 3’792’642 (2.1%) |
| *Switzerland* | 3 / 142 (2.1%) | 10'887 / 702'875 (1.6%) |
| *United Kingdom* | 0 / 7 (0%) | 128’367 / 4’771'369 (2.7%) |
| ***Overall*** | 17 / 638 **(2.7%)** | 605'925 / 26'727'952 **(2.3%)** |

**Table S4. Proportion of deaths among overall reported cases**

* Data from <https://coronavirus.jhu.edu/map.html>, 29.06.2021 (17:00, GMT)

+ The only reported patient was excluded from the analysis due to missing data on clinical course and outcome. The patient was reported during the first wave.

**Table S5. Comparison between ACHD patients and the general population**

|  | **Study population**  **(overall)** | **Study population**  **(hospitalized all)** | **Hospitalized reference population^*^** | **Study population**  **(complicated course only)** | **ICU reference population^+^** |
| --- | --- | --- | --- | --- | --- |
| **Median (IQR) age** | 34 (26-44) | 47 (35-58) | 63 (52-75) | 49 (38-59) | 63 (56-69) |
| **Female** | 52% | 47% | 40% | 42% | 19% |
| **Comorbidity** |  |  |  |  |  |
| *Renal failure* | 1.3% | 5.1% | 8.5% | 2.8% | 2.2% |
| *Cancer* | 0.5% | 2% | 6% | 2.8% | 8.3% |
| *Cardiovascular disease* | 6.3% | 12.1% | 18% | 19.4% | - |
| *Liver disease / cirrhosis* | 1.9% | 4% | 30% | 2.8% | 2.2% |
| *Immunocompromised* | 3.4% | 4% | 1.8% | 2.8% | - |
| *Lung disease* | 6.1% | 11.1% | 17.3% | 16.7% | 2.3% |
| *Diabetes* | 1.6% | 2% | 33.8% | 2.8% | 12.8% |
| *Hypertension* | 8.8% | 8.1% | 56.6% | 11.1% | 41.2% |

ICU= intensive care unit; IQR= interquartile range

*Data obtained from Richardson S, et al. Presenting Characteristics, Comorbidities, and Outcomes Among 5700 Patients Hospitalized With COVID-19 in the New York City Area. JAMA. 2020 May 26;323(20):2052-2059. doi: 10.1001/jama.2020.6775, where renal failure was referred as kidney disease; liver disease / cirrhosis as liver disease including cirrhosis and chronic hepatitis B or C; and lung disease as chronic respiratory disease including asthma, chronic obstructive pulmonary disease and obstructive sleep apnea.

+ Data obtained from Grasselli G, et al. Risk Factors Associated With Mortality Among Patients With COVID-19 in Intensive Care Units in Lombardy, Italy. JAMA Intern Med. 2020 Oct 1;180(10):1345-1355. doi: 10.1001/jamainternmed.2020.3539, where renal failure was referred as chronic kidney disease; cancer as malignant neoplasm; liver disease / cirrhosis as liver disease; diabetes as type 2 diabetes and lung disease as chronic obstructive pulmonary disease.

**Annex S1. Monthly Report**

**Clinical Outcome of COVID-19 in Adults with Congenital Heart Disease in Europe (COVID-19 Tracker)**

***Monthly Report (as per 06.05.2021)***

COVID-19 Tracker is an initiative of EPOCH ([www.sacher-registry.com/epoch](http://www.sacher-registry.com/epoch)). It reports characteristics and outcomes of cases with confirmed or suspected SARS-CoV-2 infections among adults with congenital heart disease (ACHD) from 26 tertiary care centers across Europe.

**1. Tables**

- - - - 1. **Table 1 Patient characteristics stratified by clinical course and outcome:**

Until 06^th^ of May 2021**: 629 confirmed, 65 suspected cases**

|  | **Clinical course** | | **Clinical outcome** | | | |
| --- | --- | --- | --- | --- | --- | --- |
| ***Total number = 700 patients*** | mild - moderate***^*^*** | moderate to severe - critical^+^ | no sequelae | sequelae^†^ | death | ongoing |
| ***Cardiac defect*** |  |  |  |  |  |  |
| cyanotic heart disease or ES^‡^ | 29 (74) | 9 (23) | 31 (80) | 1 (3) | 4 (10) | 3 (7) |
| Fontan physiology^‡^ | 50 (96) | 1 (2) | 48 (92) | 3 (6) | - | 1 (2) |
| TGA or truncus arteriosus^‡^ | 84 (96) | 3 (3) | 82 (93) | - | 1 (1) | 5 (6) |
| Ebstein anomaly | 13 (100) | - | 12 (92) | 1 (8) | - | - |
| coarctation (aortic)^‡^ | 57 (97) | 1 (2) | 55 (93) | 1 (2) | - | 3 (5) |
| other complex | 11 (100) | - | 10 (91) | 1 (9) | - | - |
| TOF / Pulmonary atresia^‡^ | 119 (96) | 3 (3) | 111 (90) | 3 (2) | 3 (2) | 7 (6) |
| repaired shunt lesion | 88 (97) | 3 (3) | 81 (89) | 4 (5) | 1 (1) | 5 (6) |
| repaired valve lesion^‡^ | 83 (91) | 7 (8) | 79 (87) | 1(1) | 3 (3) | 8 (9) |
| other moderate | 47 (90) | 5 (10) | 46 (88) | - | 2 (5) | 4 (7) |
| residual shunt | 19 (90) | 2 (10) | 16 (76) | 2 (10) | 1 (5) | 2 (9) |
| unrepaired valve lesion | 37 (97) | 1 (3) | 32 (84) | 2 (5) | 1 (3) | 3 (8) |
| other mild | 11 (92) | 1 (8) | 9 (75) | 1 (8) | 1 (8) | 1 (8) |
| unrepaired shunt | 4 (100) | - | 4 (100) | - | - | - |
| ***Cardiac defect complexity*** |  |  |  |  |  |  |
| mild^‡^ | 244 (95) | 13 (5) | 222 (86) | 10 (4) | 7 (3) | 19 (7) |
| moderate^‡^ | 228 (95) | 11 (5) | 218 (91) | 4 (2) | 5 (2) | 14 (6) |
| severe^‡^ | 180 (92) | 13 (7) | 176 (89) | 6 (3) | 5 (3) | 9 (4) |
| ***Female gender (%)^‡^*** | 342 (53) | 16 (43) | 316 (51) | 13 (65) | 8 (47) | 22 (52) |
| ***Age (years)*** | 35±12 | 48±12 | 35±12 | 35±12 | 52±11 | 35±10 |
| ***BMI*** |  |  |  |  |  |  |
| underweight (BMI <18) | 22 (100) | - | 19 (86) | 2 (9) | - | 1 (5) |
| normal (BMI 18-25)^‡^ | 397 (96) | 14 (3) | 386 (93) | 7 (2) | 6 (1) | 16 (4) |
| overweight (BMI 25-30) | 147 (91) | 14 (9) | 137 (85) | 7 (4) | 6 (4) | 12 (7) |
| obesity grade I (BMI 30-35) | 61 (91) | 6 (9) | 52 (78) | 4 (6) | 3 (5) | 8 (12) |
| morbid obesity (BMI > 35) | 25 (93) | 2 (7) | 22 (82) | - | 1 (4) | 4 (15) |
| ***Comorbidities*** |  |  |  |  |  |  |
| no comorbidities^‡^ | 469 (96) | 15 (3) | 440 (90) | 17 (4) | 4 (1) | 28 (6) |
| hypertension^‡^ | 57 (93) | 4 (7) | 55 (90) | 1 (2) | - | 5 (8) |
| diabetes | 11 (92) | 1 (8) | 10 (83) | - | 1 (8) | 1 (8) |
| cardiovascular disease | 33 (83) | 7 (18) | 33 (82) | 2 (5) | 5 (13) | - |
| lung disease | 36 (86) | 6 (14) | 36 (86) | - | 3 (7) | 3 (7) |
| immunocompromised | 24 (96) | 1 (4) | 21 (84) | - | 1 (4) | 3 (12) |
| cancer | 3 (75) | 1 (25) | 2 (50) | - | 1 (25) | 1 (25) |
| renal failure | 7 (88) | 1 (13) | 7 (88) | - | 1 (12) | - |
| liver disease / cirrhosis | 12 (92) | 1 (8) | 12 (93) | - | 1 (7) | - |
| ***Defect related problems*** |  |  |  |  |  |  |
| no problems^‡^ | 301 (97) | 7 (2) | 288 (93) | 9 (3) | 1 (0) | 14 (4) |
| mainly valvular problem^‡^ | 216 (95) | 10 (4) | 201 (88) | 5 (2) | 5 (2) | 17 (8) |
| mainly heart failure | 48 (87) | 7 (13) | 43 (78) | 1 (2) | 5 (9) | 6 (11) |
| mainly arrhythmia problems^‡^ | 61 (92) | 4 (6) | 58 (88) | 4 (6) | 1 (2) | 3 (4) |
| pulmonary hypertension | 24 (74) | 9 (26) | 26 (74) | 1 (3) | 5 (14) | 3 (9) |

Data are mean ± standard deviation, median (interquartile range) or number (percentage), as ES indicates Eisenmenger Syndrome; TGA indicates transposition of great arteries; TOF indicates tetralogy of Fallot; BMI indicates body mass index (in kg/m^2^).

* defined as mild (recovery at home) or moderate (in hospital stay but no ventilation, no inotropic support)

+ defined as moderate to severe (non-invasive ventilation and/or inotropic support necessary) or severe (intubation necessary) or critical (extracorporeal membrane oxygenation [ECMO] necessary)

† resolution of the infection but the patient has developed new health problems.

‡ missing value for 1 patient or more patients

**Table 2 Fatal outcomes**

| 40-50 | Male | Repaired Tetralogy of Fallot | Pre-existing severe biventricular dysfunction and progressive heart failure (had implanted CRT-D), cardiac-related liver cirrhosis and right lung hypoplasia due to an occluded right pulmonary artery. Decision regarding cardiac and liver-transplant was pending.  Admitted with ARDS. Due to comorbidities the patient was not considered a candidate for extensive cardio-respiratory support. He died at day 3 after hospital admission. | Death due to SARS-CoV-2 infection (ARDS related to COVID-19)  Underlying congenital heart disease (CHD) may have contributed to fatal outcome.  Co-morbidities/heart defect-related problems: 3 (heart failure, liver and lung disease). |
| --- | --- | --- | --- | --- |
| >60 | Male | Repaired pulmonary valve stenosis | Mild pulmonary regurgitation and acquired cardiovascular disease (coronary artery disease, s/p ischemic stroke, abdominal aneurysm, atrial fibrillation) and COPD. NYHA class II prior to COVID-19.  Admitted with bilateral pneumonia leading to ARDS requiring intubation at the day of admission. Renal failure occurred 3 days after presentation. He died at day 11 after admission with multiorgan failure. | Death due to SARS-CoV-2 infection (ARDS related to COVID-19)  Underlying CHD unlikely to contribute to fatal outcome.  Co-morbidities/heart defect-related problems: 3 (previous stroke, coronary artery disease, lung disease) |
| 40-50 | Female | Bicuspid aortic valve with severe aortic stenosis | Presentation with decompensated heart failure due to severe aortic stenosis, requiring urgent surgical aortic valve replacement. Complicated postoperative course with cardiogenic shock requiring veno-arterial ECMO.  Developed ARDS on first postoperative day and tested positive for SARS-CoV-2. She died 7 days after surgery. | Death with SARS-CoV-2 infection; (Postoperative death due to heart failure)  Underlying CHD was the main reason for the fatal outcome.  Co-morbidities/heart defect-related problems: 1 (heart failure) |
| 50-60 | Female | Eisenmenger syndrome with unrepaired complete atrioventricular septal defect | Severe pulmonary hypertension, heart failure and moderate leucopenia. Presentation at the emergency department with bilateral pneumonia and ARDS.  Due to her functional status (NYHA class III) prior to COVID-19 and personal preferences, she was transferred to a palliative care center. She died at day 32 after initial hospital admission. | Death due to SARS-CoV-2 infection (ARDS related to COVID-19)  Underlying CHD contributed to fatal outcome.  Co-morbidities/heart defect-related problems: 3 (trisomy 21, heart failure, pulmonary hypertension) |
| 40-50 | Female | Eisenmenger syndrome with unrepaired complete atrioventricular septal defect | Severe pulmonary hypertension, heart failure and severely reduce renal function. Presentation at the emergency department with ARDS.  Due to her functional status (NYHA class IV) prior to COVID-19 and personal preferences, she was discharged home. She died at home 22 days after initial hospital presentation. | Death due to SARS-CoV-2 infection (ARDS related to COVID-19)  Underlying CHD contributed to fatal outcome.  Co-morbidities/heart defect-related problems: 4 (trisomy 21, heart failure, pulmonary hypertension, kidney failure) |
| 50-60 | Male | Partial anomalous pulmonary venous drainage  Persistent foramen ovale (PFO) with severe right-to-left shunt | History of type 2 diabetes mellitus and esophageal cancer. Incidental diagnosis of partial anomalous pulmonary venous drainage during the diagnostic cancer workup. Normal right ventricular dimensions, no evidence of pulmonary hypertension.  Hospital admission for elective esophagectomy. Recurrent postoperative hypoxemia requiring re-intubation. Diagnosed with COVID-19 on postoperative day 4. Subsequently severe ARDS with hemodynamic instability, severe pulmonary hypertension and multiple secondary infectious complications. Diagnosis of a patent foramen ovale with severe right-to-left shunting on postoperative day 23. Emergent veno-arterial ECMO on postoperative day 26 and percutaneous PFO closure on postoperative day 27. Weaning from ECMO 7 days after PFO-closure. The patient recovered, was discharged and died during inpatient rehabilitation. | Death still unclear  Admission for noncardiac surgery  Patent foramen ovale was a contributor to complicated disease course. Partial anomalous pulmonary venous return likely not substantially contributing to disease course.  Co-morbidities/heart defect-related problems: 2 (diabetes, cancer) |
| 40-50 | Male | Eisenmenger due to unrepaired VSD | Patient institutionalized at the time of COVID-19 diagnosis. Initially treated at his institution with supplemental oxygen and subcutaneous morphine. Referral to his regional hospital of reference due to impaired consciousness secondary to morphine intoxication in the context of worsened renal failure. Progressive respiratory failure over time, probably due to bacterial pulmonary superinfection. Patient/family did not desired invasive mechanical ventilation. Therefore, a palliative approach was stablished. The patient deceased shortly thereafter. | Death due to SARS-CoV-2 infection  Underlying CHD contributed to death  Co-morbidities/heart defect-related problems: 2 (Trisomy 21, renal failure stage IIIb) |
| 40-50 | Male | Repaired AVSD with mechanical mitral valve prosthesis.  Thranscatheter aortic coarctaction repair before AVSD surgery | No significant complications before admission. Admitted to hospital because of endocarditis of mitral valve prosthesis complicated with an ischemic stroke. Positive PCR for Sars-Cov2 on admission screening. Intubated and on inotropic drugs for almost 2 months due to multisystem organ failure and ARDS. | Death with SARS-CoV-2 infection; (Multisystem organ failure and ARDS).  Underlying complication of CHD was the main reason for the fatal outcome.  Co-morbidities/heart defect-related problems: 1 (immunocompromised: IgG4 related disease on corticosteroids) |
| 50-60 | Male | Repaired ventricular septal defect.  Bio-aortic valve replacement (3x) due to endocarditis. Residual moderate paravalvular aortic valve regurgitation, aortic root aneurysm. | History of recurrent infective endocarditis (3x). Arterial hypertension. Institutionalized due to his psychiatric history (bipolar disorder). Recurrent episodes of sustained ventricular tachycardia. ICD implantation discussed 1 year ago with health care proxy but finally not performed due to fear of inappropriate shocks. Recurrent hospitalisations for decompensated heart failure with preserved ejection fraction. The patient tested positive for SARS-CoV-2 infection at his institution during a general outbreak. He presented mild symptoms (fever) and was already recovering 4 days after the diagnosis. No signs or symptoms of decompensated heart failure during convalescence. Sudden death during the night. | Death with SARS-CoV-2 infection; cause unclear; probably related to arrhythmia.  Underlying CHD and acquired severe aortic regurgitation were probably the main reasons for the fatal outcome.  Co-morbidities/heart defect-related problems: 2 (arterial hypertension, heart failure) |
| 40-50 | Female | Severe mitral valve regurgitation due to parachute mitral valve | Patient lost to follow-up since 2016. History of congestive heart failure secondary to severe mitral valve regurgitation due to parachute mitral valve. She was completely dependent for activities of daily living due to cerebral palsy with severe psychomotor retardation and epilepsy. No radiological signs of pneumonia at diagnosis. She stayed at home during convalescence. She died two months and eight days after COVID-19 diagnosis due to progressive deterioration of her overall medical condition (dehydration and anorexia). | Death after SARS-CoV-2 infection; cause unclear. Respiratory failure due to worsening of chronic heart failure in the context of progressive deterioration of her overall medical condition was postulated.  Underlying CHD likely to contribute to fatal outcome.  Co-morbidities/heart defect-related problems: 2 (cerebral palsy, heart failure) |
| 20-30 | Female | Repaired AVSD with mechanical mitral valve prosthesis | Anticoagulation with subcutaneous low-molecular-weight heparin due to mechanical mitral valve prosthesis. Otherwise healthy woman at 29 weeks of pregnancy. Hospital admission due to respiratory failure 18 days after diagnosis of COVID-19, for which she was treated with steroids at home until the day before admission. At admission, a transthoracic echocardiography showed normal biventricular function and pronounced D-shaping with concomitant obstruction of the LVOT. Rapid transfer to the ICU due to refractory respiratory insufficiency and hypotension, where invasive mechanical ventilation was started. Legionella urinary antigen testing was twice positive. Broad-spectrum antimicrobial treatment was started. A transesophageal echocardiography ruled-out a thrombosis of the mechanical mitral valve prosthesis. An emergency caesarean section was successfully performed. During surgery, no relevant blood loss occurred. Rapid deterioration of the respiratory and hemodynamic situation back at the ICU with refractory shock and multiorgan failure. The patient died the day after admission. | Death due to SARS-CoV-2 infection (ARDS related to COVID-19-related bacterial superinfection)  Underlying congenital heart disease (CHD) may have contributed to fatal outcome.  Co-morbidities/heart defect-related problems: 0 |
| 40-50 | Female | Cor triatriatum sinistrum, bicuspid aortic valve and VSD  VCD closure at 5 y.o  Intra atrial membrane resection at 43 y.o | History of severe asthma, Type 2 diabetes mellitus and renal dysfunction (GFR= 37 mL/min/1.73m2). Transferred for cardiovascular rehabilitation after hospital admission for heart failure and atrial arrhythmia. Admitted to the intensive care four weeks after discharge due respiratory failure in the context of bilateral pneumonia related to COVID-19. She died 10 days after admission in the ICU due to multisystem organ failure and ARDS | Death due to SARS-CoV-2 infection (ARDS related to COVID-19)  Underlying congenital heart disease (CHD) may have contributed to fatal outcome.  Co-morbidities/heart defect-related problems: 7 (heart failure, atrial arrhythmia, renal dysfunction, diabetes, hypertension, asthma, obstructive sleep apnea). |
| >60 | Male | ASD II diagnosed late in life due to pulmonary embolism.  Closure due to missing hemodynamic significance and elevated arterial pulmonary pressure/restrictive RV not performed. | Presentation with worsening dyspnea. COVID-19 was diagnosed upon admission. A treatment with Remdesivir and Dexamethason were started. Referral to the intensive care unit for non-invasive ventilation due to progressive hypoxemia. Rapid deterioration with respiratory insufficiency and ARDS. Because of his general condition and multiple co-morbidities, invasive mechanical ventilation was discard. The patient died shortly thereafter. | Death due to SARS-CoV-2 infection (ARDS related to COVID-19).  Underlying CHD and specially age co-morbidities (overall general condition) contributed to the fatal outcome.  Co-morbidities/heart defect-related problems: 5 (diabetes, cerebrovascular disease, multiple thromboembolic episode, chronic hypoxemia, hypertension). |
| 50-60 | Male | Unrepaired L-TGA, complete heart block | Patient with history of systemic right ventricular dysfunction, PM for complete heart block and permanent atrial arrhythmia. Diagnosis of pre and post-capillary arterial hypertension during heart transplantation work-up (PVR= 8.82 Wood units) contraindicating heart transplantation. Hospitalized for cardiogenic shock two months after CRT-D implantation requiring cardio-pulmonary support. COVID-19 during hospital stay with rapid hemodynamic deterioration. A palliative approach was stablished. The patient deceased shortly thereafter. | Death due to cardiogenic shock related to advance heart failure and COVID-19 pneumonia.  Underlying CHD contributed to the fatal outcome  Co-morbidities/heart defect-related problems: 1 (heart failure) |
| 30-40 | Female | Eisenmenger syndrom due to unrepaired VSD | Presentation with worsening dyspnea, severe cyanosis and hemoptysis. COVID-19 was diagnosed upon admission. Due to her functional status, the patient was included in a palliative care program. She deceased 2 days after admission. | Death due to SARS-CoV-2 infection (ARDS related to COVID-19)  Underlying congenital heart disease have contributed to fatal outcome.  Co-morbidities/heart defect-related problems: 2 (Trisomy 21, pulmonary hypertension) |
| >60 | Male | Repaired Tetralogy of Fallot  RV-PA conduit implantation in 2005 and replacement in 2021 | Irregular follow-up with history of severely reduce right ventricular systolic function due to severe RV-PA conduit stenosis and right pulmonary artery stenosis. Admission for elective replacement of the RV-PA conduit and patch augmentation of the right pulmonary artery. Prolonged postoperative convalescence due to right heart failure. Nosocomial COVID-19 during hospital stay with rapid development of severe bilateral pneumonia with rapid progression to ARDS, bacterial superinfection, multisystem failure and death. | Death due to SARS-CoV-2 infection (ARDS related to COVID-19-related bacterial superinfection)  Underlying congenital heart disease (CHD) may have contributed to fatal outcome.  Co-morbidities/heart defect-related problems: 1 (heart failure) |
|  |  |  |  |  |

**2. Graphics**

**2.1. Figure 1**

Distribution of cumulative cases stratified by outcome (recovery, still ongoing and death).
